# Defining the relationship between the hip, pelvis, and lumbar spine

**DOI:** 10.1016/j.xnsj.2026.100883

**Published:** 2026-04-01

**Authors:** Emily S. Mills, Mary K. Richardson, Jennifer C. Wang, Brian C. Chung, Melissa Romoff, Nathanael D. Heckmann

**Affiliations:** a Department of Orthopaedic Surgery, Keck School of Medicine of USC, 1200 N State St GH 3900, Los Angeles, CA 90033, United States; b Department of Orthopaedic Surgery, University of California, Irvine, School of Medicine, 101 The City Dr S, Pavilion 3, Building 29A, Orange, CA, United States

**Keywords:** Hip-spine biomechanics, Lumbosacral fusion, Pelvic femoral angle, Sagittal Balance, Spinopelvic Alignment, Standing alignment

## Abstract

**Introduction:**

While the hip, pelvis, and lumbar spine are known to move synchronously, their exact relationship has yet to be determined. The aim of this study was to define the biomechanical relationship between the hip, pelvis, and lumbar spine.

**Methods:**

A cohort of fifty healthy subjects between 18- and 35-years-old were recruited to participate, including 25 males and 25 females. Exclusion criteria included presence of back or hip pain, inability to stand upright or sit with hips flexed to 90°, history of ankylosing spondylitis, femoroacetabular impingement, or prior hip or spine surgery. Three lateral radiographs were obtained for each patient, including standing, relaxed seated, and the flexed-forward seated views. Radiographic variables measured included sacral slope (SS), pelvic-femoral angle (PFA), and lumbar lordosis (LL). Calculations included the change in SS (ΔSS), the change in PFA (ΔPFA), the change in LL (ΔLL). Pearson correlations were used to determine the relationship between ΔSS, ΔPFA, and ΔLL with significance set to p<.05.

**Results:**

The average age of participants was 25.70±2.34 years with a body mass index (BMI) of 24.10±3.02 kg/m^2^. For every degree increase of hip flexion, lumbar flexion decreased 0.84° (R=-0.75, p<.001). Similarly, ΔPFA was strongly negatively correlated with ΔSS, with every degree increase of ΔPFA yielding a decrease of 0.71° in ΔSS (R=-0.85, p<.001). ΔSS was strongly correlated with the ΔLL. For every degree that ΔLL increased, ΔSS increased 0.63° (R=0.85, p<.001).

**Conclusions:**

Motion between the hip, pelvis, and lumbar spine is highly correlated. For every degree increase of hip flexion, the pelvis tilts posteriorly 0.71, causing the lumbar spine to flex 1.58 degrees to maintain upright posture. For every degree increase of hip flexion, the lumbar spine decreases flexion 0.86 degrees. Given the increased interest and clinical applicability of spinopelvic biomechanics in the spine community, these definitions are essential to moving forward with research in this topic.

## Introduction

Hip spine syndrome was first described by Offierski and MacNab in 1983 in a landmark study that described the overlapping symptoms of hip and lumbar spine disease [1]. Studies have since attempted to delineate the relationship between the lumbar spine and the hip. In the arthroplasty literature, it has been well established that lumbar fusion increases dislocation rate [[2], [3], [4], [5], [6], [7]]. This has been theorized to be because of two mechanisms. First, the lumbar spine may be fused in a standing position, which does not allow the pelvis to tilt anteriorly while sitting. This does not allow increased anteversion of the acetabulum and therefore predisposes to posterior dislocation [8]. Second, as the lumbar spine and hip work together to flex through the pelvis when sitting down, decreased motion of the lumbar spine has been theorized to increase flexion through the hip [9]. Therefore, as lumbar motion decreases, hip motion is thought to increase, leading to an increased risk of dislocation. However, this direct relationship has yet to be shown.

Treatment of hip disease has reciprocally been found to decrease back pain, with one study finding improved spine patient reported outcome measures (PROMs) in patients after undergoing total hip arthroplasty (THA) [10]. Additionally, timing of THA has been shown to affect lumbar fusion outcomes, with patients undergoing THA prior to lumbar fusion shown to have decreased rates of adjacent segment disease and pseudarthrosis compared to those who undergo THA following lumbar fusion [11]. These studies indicate that increased motion through the hip places less stress through the lumbar spine and therefore improves back pain as well as outcomes following fusion.

While studies such as those mentioned above have shown a relationship between the lumbar spine and the hip, it has yet to be defined biomechanically. Many studies assess the relationship between the lumbar spine and the hip when transitioning from a standing to a sitting position, with the majority of the literature using sacral motion as a surrogate for spinal motion [9,[12], [13], [14]]. While a relationship between the 2 has been assumed because the sacrum is part of the spine, the sacrum is fused to the pelvis and therefore is only a true measure of pelvic motion. Sacral motion is currently used interchangeably in the literature to define pelvic motion, sacral motion, spinopelvic motion, and spinal motion, bringing unnecessary confusion to an already complex topic. It is therefore critical to properly define these terms and to determine the relationships among contributors to pelvic motion. As such, the purpose of this study was to delineate the relationship between the lumbar spine, pelvis, and hip by assessing their biomechanical relationship when moving from standing to seated positions. Secondarily, we aimed to determine if there is a variability throughout the population in segmental motion of the lumbar spine.

## Methods

### Data collection

**Fig. 1:**
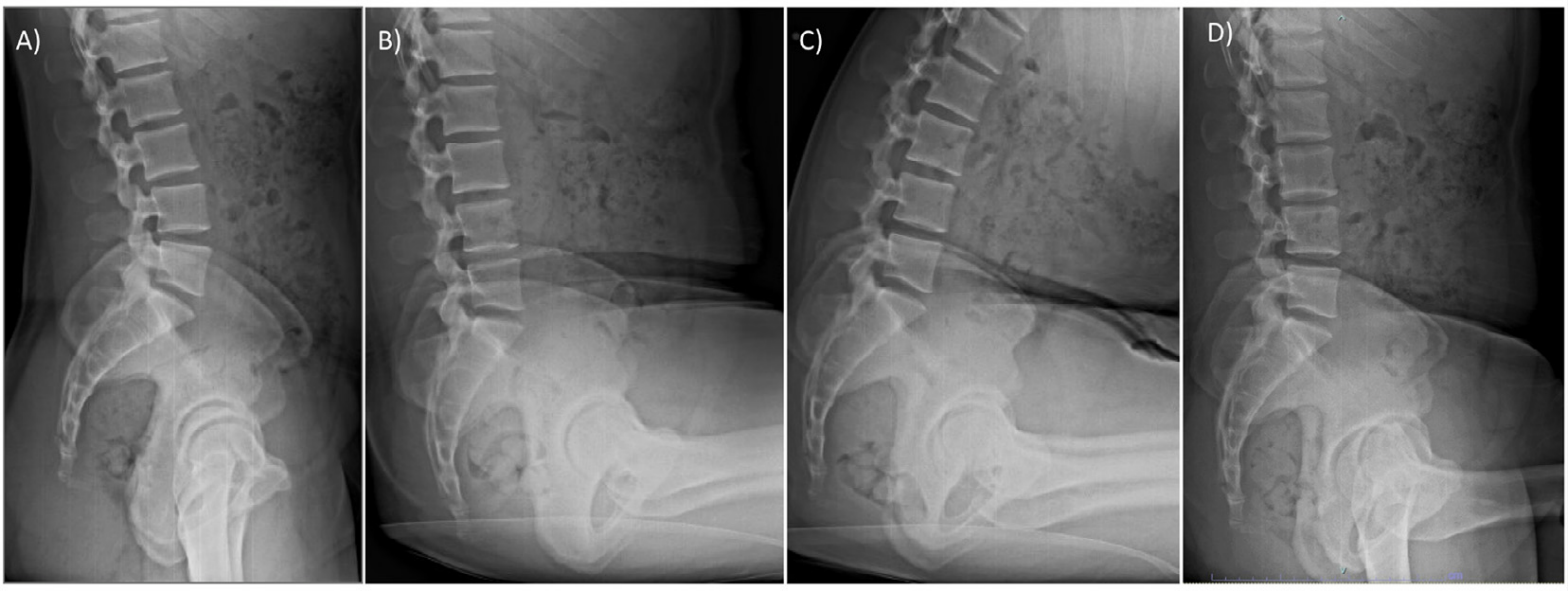
(A) Standing (B) Sitting, (C) Flexed forward, and (D) Single-leg step up radiographs.

Following institutional review board approval (IRB: HS-20-00874), 50 healthy subjects, 25 male and 25 female, were recruited to participate from June 2021 to August 2022. Inclusion criteria included age between 18- and 35-years old. Exclusion criteria included presence of back or hip pain, inability to stand upright or sit with hips flexed to 90°, history of ankylosing spondylitis, femoroacetabular impingement, or prior hip or spine surgery. The goal of these criteria was to ensure a healthy cohort free of pathology or conditions altering spinopelvic motion. Female patients were provided a urine pregnancy test prior to participation. Those with a positive test were excluded. Lateral plain radiographs in 3 postural positions simulating common daily positions were obtained for each participant: (1) standing, (2) relaxed-seated (to simulate sitting down on a chair), and (3) flexed-seated (to simulate putting on socks and shoes) (Fig. 1). Radiographs included the superior endplate of L1 proximally and the femoral shafts distally.

### Measurements and calculations

An experienced physician-researcher completed all fifty patients’ radiographic measurements. Each radiographic variable was measured twice. If there was less than 5° difference between the 2 values, the values were averaged to obtain the final value. If there was more than 5 degrees of variability, the value was measured for a third time to recheck validity of the first 2 values and the third measurement replaced the inaccurate initial value. After the final values were calculated, all outliers were confirmed with repeat measurements to further ensure reliability. Radiographic variables measured included sacral slope (SS), pelvic-femoral angle (PFA), and lumbar lordosis (LL), pelvic incidence (PI), pelvic tilt (PT), lumbar distribution index (LDI), and the angle between each consecutive superior and inferior endplates (L1–2, L2–3, etc.) to calculate segmental lordosis (SL).

Subsequently, calculations included the change in SS (ΔSS), the change in PFA (ΔPFA), the change in LL (ΔLL), and the change in SL (ΔSL) when going from (1) a standing to relaxed seated position and (2) a standing to flexed-forward seated position. Relationships between ΔSS, ΔPFA, and ΔLL were defined using motion from a standing to flexed-forward seated position, as that is when maximal motion occurs.

### Outcomes

The primary outcome of this study was the correlation between hip, pelvic, and spine motion. Secondary outcomes included (1) the percentage of participants with the majority of motion from the upper lumbar segments in both the standing to relaxed-seated motion and the standing to flexed-forward motion, and (2) standing radiographic parameters that correlated with the lumbar segment of maximal motion.

### Statistical analyses

Patient demographics (e.g., age, height, weight, and body mass index [BMI]), descriptive variables, radiographic measurements, and calculations were presented as means with standard deviations and ranges, or percentages where appropriate to establish reference values. Pearson correlations were used to determine the relationship between ΔSS, ΔPFA, and ΔLL as well as the correlation between standing parameters and the lumbar segment of maximal motion. All statistical analyses were performed using SPSS version 28 (IBM, Armonk, NY, USA), with significance set to p<0.05.

## Results

Mean standing PT was 13.1°±7.7° (range − 7°−31°), mean standing PFA was 185.4°±6.8° (range 169°−211°), mean standing LL was 53.3°±10.8° (range 26°−71°), and mean PI was 49.3°±11.4°. Mean sitting PT was 29.7°±10.0° (range 10°−50°), mean sitting PFA was 114.1°±9.6° (range 98°−135°), and mean sitting LL was 24.8°±11.7° (range − 2°−47°) (Table 1).

**Table 1:** Average radiographic measurements by position.

Parameter	Standing			Relaxed-Seated			Flexed-Seated		
	Mean (°)	SD (°)	Range	Mean (°)	SD (°)	Range	Mean (°)	SD (°)	Range
PI	49.3	11.4	24, 72	-	-	-	-	-	-
SS	36.2	8.4	16, 53	19.5	9.1	0, 42	51.4	11.9	20, 84
PT	13.1	7.7	−7, 31	29.7	10.0	10, 50-	−2.1	12.4	−30, 31
PFA	185.4	6.8	169, 211	114.1	9.6	98, 135	85.3	11.3	57, 110
LL	53.3	10.7	28, 71	24.6	11.9	−2, 47	−7.7	11.5	−31, 24
L1–L2	5.6	2.5	1, 11	5.4	3.0	−3, 10	−1.8	2.2	−7, 3
L2–L3	7.7	3.3	1, 13	5.2	4.1	−5, 13	−3.0	2.6	−9, 2
L3–L4	11.2	3.4	4, 19	4.4	3.7	−3, 12	−2.5	2.6	−9, 4
L4–L5	14.2	3.6	4, 23	3.3	3.1	−4, 11	−2.2	3.6	−10, 6
L5–S1	13.4	4.8	3, 24	6.0	3.7	−3, 14	0.8	4.7	−8, 12

PI, pelvic incidence; SS, sacral slope; PT, pelvic tilt; PFA, pelvic femoral angle; LL, lumbar lordosis; SD, standard deviation.

### Standing to sitting motion

From the standing to the relaxed-seated position, subjects had an average ΔSS of 16.7°±8.5° (range − 2°−36°), ΔPT of − 16.7°±8.5° (range − 36°−2°), ΔPFA of 71.3°±10.1° (range 50°−93°), ΔLT of − 3.8°±5.3° (range − 14°−4°). Participants had an average ΔLL of 28.5°±11.6°, ΔL1–L2 of 0.24°±4.0°, ΔL2–L3 of 2.5°±4.0°, ΔL3–L4 of 6.9°±3.5°, ΔL4–L5 of 10.9°±3.9°, and ΔL5–S1 of 7.3°±5.5°. The segment with the greatest motion from standing to relaxed-seated was L4–L5 in 26 (52%), L5–S1 in 13 (26%), and L3–L4 in 11 (22%) volunteers. Eleven (22%) of subjects had the majority of motion through L1–L4, and 39 (78%) had the majority of motion throughout the L4–S1 segments (Table 2).

**Table 2:** The number of subjects who had maximal motion at each level when transitioning from the standing to relaxed-seated position and when transitioning from the standing to flexed-seated position.

	Count
**Segment of maximal motion from standing to relaxed-seated**	N (%)
**L1–2**	0 (0%)
**L2–3**	0 (0%)
**L3–4**	11 (22%)
**L4–5**	26 (52%)
**L5–S1**	13 (26%)
**L1–4**	11 (22%)
**L4–S1**	39 (78%)
**Segment of maximal motion from standing to flexed–seated**	
**L1–2**	0 (0%)
**L2–3**	3 (6%)
**L3–4**	10 (20%)
**L4–5**	24 (48%)
**L5–S1**	8 (16%)
**L3–4 and L4–5**	2 (4%)
**L4–5 and L5–S1**	2 (4%)
**L2–3 and L4–5**	1 (2%)
**L1–4**	28 (56%)
**L4–S1**	22 (44%)

### Standing to flexed forward motion

From the standing to flexed-forward position, subjects had an average ΔSS of − 15.2°±9.7° (range − 39°−7°), ΔPT of 15.2°±9.7° (range − 7°−39°), and ΔPFA of 100.1°±10.8° (range 78°−126°). Participants had an average ΔLL of 61.12°±10.3° (range 42°−79°), ΔL1–L2 of 7.4°±2.3° (range 1°−12°), ΔL2–L3 of 10.7°±3.7° (range 2°−19°), ΔL3–L4 of 13.78 °±3.0° (range 6°−22°), ΔL4–L5 of 16.4°±4.2° (range 5°−26°), and ΔL5–S1 of 12.6°±5.8° (2°−22°).

**Fig. 2:**
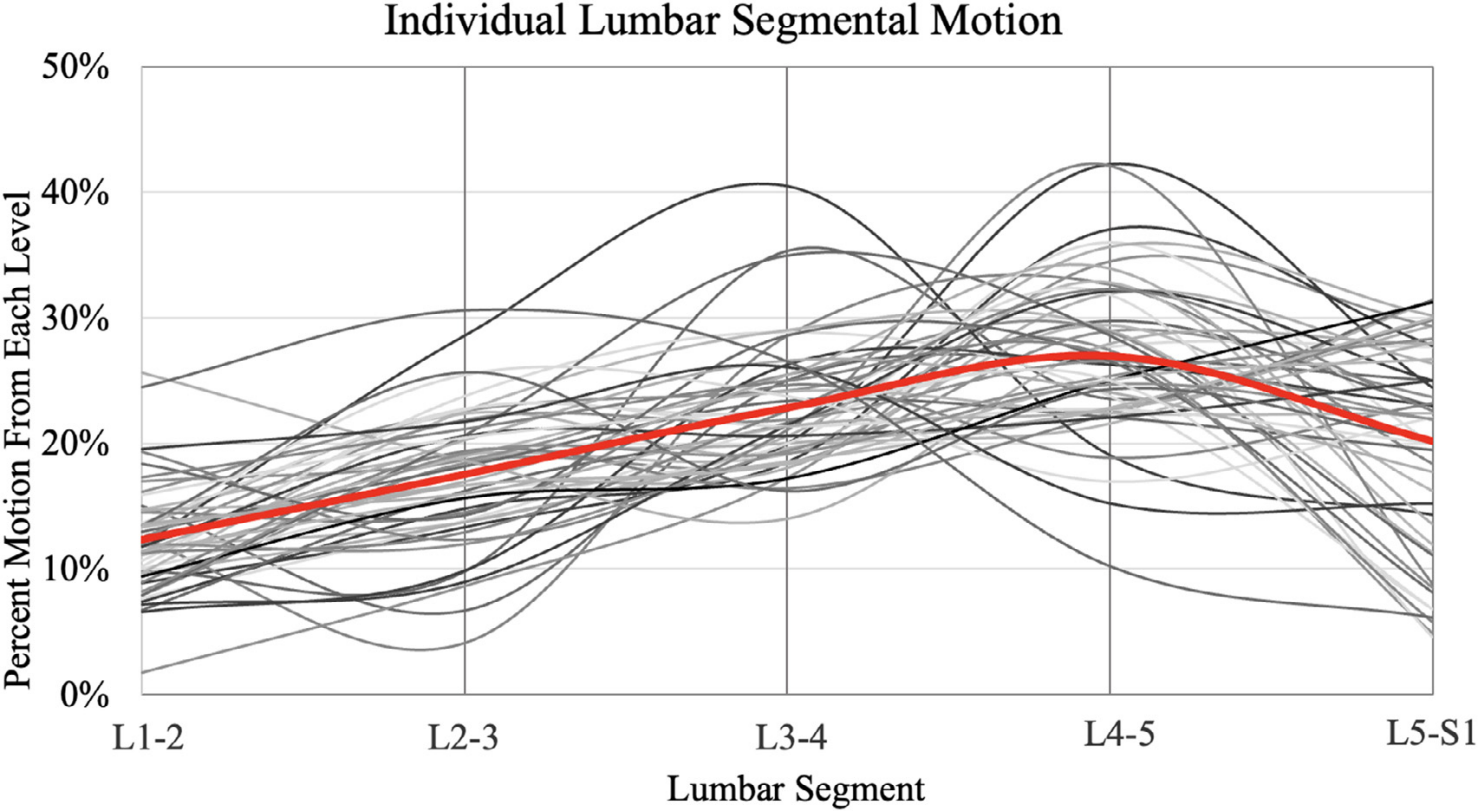
The variability in lumbar motion when going from standing to the flexed forward position. The 50 subjects are each represented by a black line. The average is shown in red.

During the standing to flexed-forward seated position, maximal motion occurred at L4–L5 in 24 participants (48.0%), L3–L4 in 10 (20.0%), L5–S1 in 8 (16.0%), and L2–L3 in 3 (6.0%). Maximal motion occurred at both L3–4 and L4–5 in 2 participants (4.0%), L4–5 and L5–S1 in 2 (4.0%), and L2–3 and L4–5 in one (2.0%) subject. Twenty-four (48.0%) volunteers had > 50% of motion through the lower lumbar segments (L4–S1), and the remaining 26 participants (52.0%) had the majority of motion through the upper lumbar segments (L1–L4) (Fig. 2).

### Relationship between the hip, pelvis, and lumbar spine

**Fig. 3:**
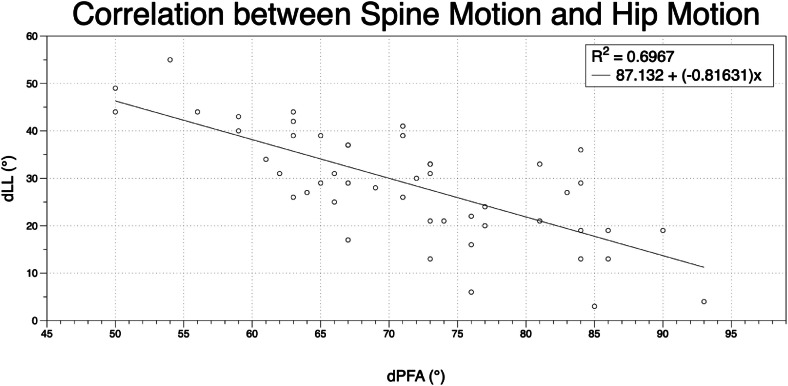
Correlation between spine motion and hip motion when going from a standing to a flexed forward position. dLL = delta lumbar lordosis when going from a standing to a flexed forward position; dPFA = delta pelvic femoral angle when going from a standing to a flexed forward position.

**Fig. 4:**
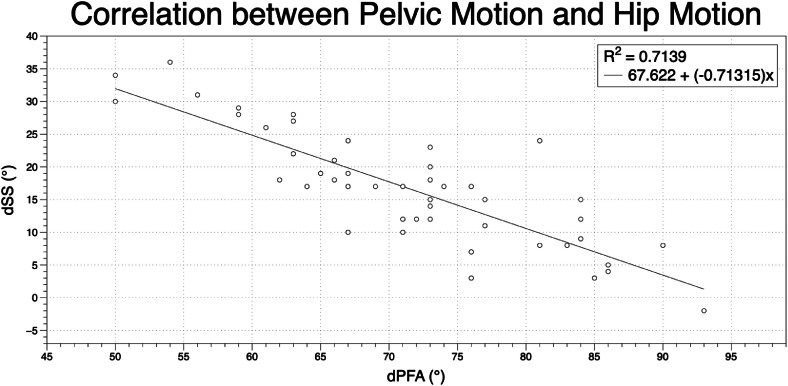
Correlation between pelvic motion and hip motion when going from a standing to a flexed forward position. dSS = delta sacral slope when going from a standing to a flexed forward position; dPFA = delta pelvic femoral angle when going from a standing to a flexed forward position.

**Fig. 5:**
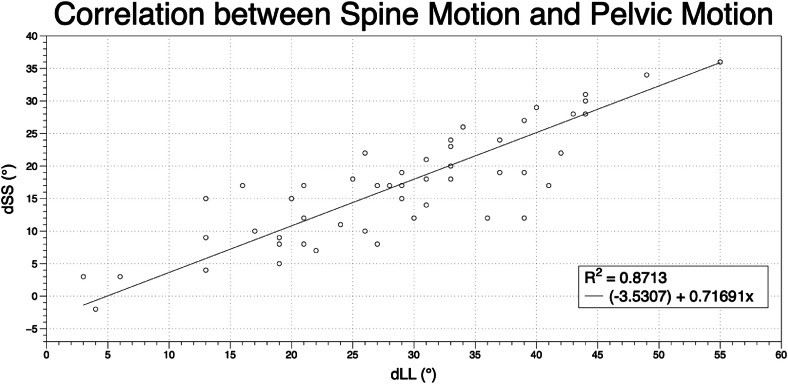
Correlation between spine motion and pelvic motion when going from a standing to a flexed forward position. dSS = delta sacral slope when going from a standing to a flexed forward position; dLL = delta lumbar lordosis when going from a standing to a flexed forward position.

When transitioning from a standing to a flexed-forward seated position, there was a strong negative correlation between ΔPFA and ΔLL. For every degree increase of hip flexion, lumbar flexion decreased 0.84° (R=−0.75, p<0.001; Fig. 3). Similarly, ΔPFA was strongly negatively correlated with ΔSS, with every degree increase of ΔPFA yielding a decrease of 0.71° in ΔSS (R=−0.85, p<.001; Fig. 4). ΔSS was strongly correlated with the ΔLL. For every degree that ΔLL increased, ΔSS increased 0.63° (R=0.85, p<.001; Fig. 5).

### Predictors of lumbar segmental motion

When assessing whether standard radiographic measurements are predictive of motion, the L5–S1 segment was most likely to be predicted by these variables, with PI, standing PT, and standing PFA all moderately negatively correlated with motion at the segment (R=−0.42, −0.44, and − 0.43, respectively, p<.05) (Table 3).

**Table 3:** Correlations of standing parameters and motion through each lumbar segment, as well as motion through upper (L1–L4) and lower (L4–S1) segments.

	ΔL1–L2		ΔL2–L3		ΔL3–L4		ΔL4–L5		ΔL5–S1		ΔL1–L4		ΔL4–S1	
	Pearson	p-value	Pearson	p-value	Pearson	p-value	Pearson	p-value	Pearson	p-value	Pearson	p-value	Pearson	p-value
**PI**	0.103	.477	0.165	.252	0.295	**.037**	−0.048	.743	−0.419	**.002**	0.285	**.045**	−0.362	**.010**
**SS**	−0.044	.760	0.296	**.037**	0.150	.298	−0.242	.091	−0.17	.239	0.217	.130	−0.280	**.049**
**PT**	0.200	.163	−0.078	.591	0.273	.055	0.193	.180	−0.435	**.002**	0.185	.199	−0.231	.107
**PFA**	0.239	.094	−0.051	.725	0.186	.197	0.043	.766	−0.426	**.002**	0.172	.233	−0.313	**.027**
**LL**	−0.035	.807	0.283	**.046**	0.153	.287	−0.258	.070	−0.158	.273	0.216	.133	−0.281	**.048**

Bold denotes significance. Δ = delta from standing to flexed-seated.

When assessing motion through upper versus lower lumbar segments, PI had a low positive correlation with motion through L1–L4 (R=0.29, p<.05). PI and standing PFA had moderate negative correlations to motion through L4–S1 (R=−0.36, R=−0.31, respectively, all p<0.05), and SS and LL had low negative correlations to motion through L4–S1 (R=−0.28, R=−0.28, respectively, both p<.05). There was a moderate correlation between segment of maximal lordosis while standing and segment of maximal motion (R=0.40, p<.05). Additionally, the segment of maximal lordosis while standing was most often the segment of maximal motion (27 subjects, 54%). There was no correlation between the segment of maximal motion when transitioning from a standing to relaxed-seated position and the segment of maximal motion when transitioning from standing to flexed-seated position (R=0.27, p>.05) (Table 3).

## Discussion

Here, we provide important realizations and clarifications regarding normative spinopelvic biomechanics. When transitioning from a standing to a flexed-forward sitting position, the pelvis tilts posteriorly to accommodate for hip flexion. For every degree increase of hip flexion, the pelvis tilts posteriorly 0.71. The lumbar spine then flexes to maintain sagittal balance, as shown by the strong correlation between ΔSS and ΔLL. For every degree increase of posterior pelvic tilt, the lumbar spine flexes 1.58 degrees. We have additionally confirmed that there is a strong negative linear relationship between hip flexion and lumbar flexion. For every degree increase of hip flexion, the lumbar spine, on average, decreases flexion 0.86 degrees. While these relationships have been previously assumed, they have not been numerically defined prior to the current study.

Our data has important implications for both arthroplasty and spine surgeons. Arthroplasty surgeons frequently use the change in sacral slope as a measure of spinal motion. Ike et al. first classified spinal motion using pelvic tilt, and then went on to reclassify using sacral slope [12]. A large portion of the arthroplasty community currently uses ΔSS as a measure of spinal motion, with ΔSS<10 degrees classified as a stiff spine, ΔSS 10–30 classified as a normal spine, and ΔSS > 30 classified as a hypermobile spine [15]. In this study, we found that ΔSS is strongly correlated with ΔLL, meaning that when the sacrum (i.e., pelvis) retroverts, the lumbar spine flexes. It is important to recognize the difference between the 2, however. The sacrum is fused to the pelvis, and therefore, ΔSS is only a true measure of pelvic motion. When the pelvis retroverts, the lumbar spine flexes to maintain an upright posture. Here, we found that for every degree the pelvis retroverts, the lumbar spine flexes 1.58° with a strong correlation.

Similarly, we found a strong negative correlation between the lumbar spine and hip flexion. For every degree increase of hip flexion, lumbar flexion decreased 0.86°. Previously, it has been theorized that the hip and lumbar spine both contribute to flexion through the pelvis when transitioning from a standing to sitting position [16]. Here, we have quantified this relationship. Motion through the lumbar spine is necessarily lost after fusion and is likely transferred to adjacent segments [17]. Therefore, it may be beneficial to find ways to decrease lumbar motion prior to and following lumbar fusion to decrease mechanical complications, such as adjacent segment disease and pseudarthrosis. As hip osteoarthritis (OA) has been shown to decrease hip motion [18] and total hip arthroplasty (THA) has been shown to increase hip motion [12], it may be advantageous to recommend THA prior to lumbar spinal fusion if a patient has concomitant hip OA. Previous studies found a decreased risk of adjacent segment disease and revision in patients undergoing THA prior to lumbar fusion compared to those undergoing THA after lumbar fusion [11,19]. This phenomenon may be explained by the results here. However, further research is necessary to determine the benefits of increased hip motion on lumbar fusion outcomes.

This data additionally gives new insight into normal motion through the lumbar spine. Previously, it was thought that the lower lumbar segments are responsible for the majority of lumbar motion in everyone [[20], [21], [22], [23], [24], [25]]. Hey et al. studied lumbar motion in 70 patients with acute back pain and found that, on average, the L4–5 segment had the most mobility followed by the L5–S1 segment [21]. Similarly, Bae et al. assessed lumbar motion from standing to various sitting positions and concluded that the L4–5 and L5–S1 segments contributed most to lumbar motion [23]. From these studies, it has been assumed that the L4–L5 and L5–S1 segments contribute most to motion in the entire population. While our findings show that the L4–5 segment commonly contributes the most to lumbar motion, there is significant variability. We found that L2–3 and L3–4 are the maximal motion segments in 6.0% and 20.0% of people, respectively. Furthermore, 20.0% of volunteers had the majority of motion through the upper lumbar segments (L1–L4) when transitioning from a standing to relaxed-sitting position, and 56.0% had the majority of motion through the upper lumbar segments when transitioning from a standing to flexed-seated position.

This may have important ramifications in terms of lumbar fusion. If a patient has maximal motion through the L3–L4 segment and minimal motion through the L5–S1 segment, fusing the L5–S1 will likely not cause an alteration to the natural biomechanics. Alternatively, if a patient has maximal motion at the L5–S1 segment, fusion of this level will likely greatly alter the natural motion through the spine. Additionally, fusion of the L4–S1 segments in patients who have the majority of motion through the upper lumbar segments may not affect lumbar biomechanics in the same way as if those segments are fused in patients who have the majority of motion through the lower lumbar segments. While not included in the present study, attention to individualized preoperative motion may yield prediction of postoperative mechanical complications, such as pseudarthrosis, rod fracture, and adjacent segment disease. It therefore may be advisable to obtain dynamic radiographic studies prior to fusion for this reason. In this study, sitting views were compared to standing views as a measure of dynamic motion that is applicable to everyday life. Several other authors have found that sitting and standing views better capture spinal instability as compared to traditional flexion/extension views [26,27] and have moved towards obtaining sitting and standing views instead.

Despite these findings, several limitations must be acknowledged. This study evaluated a young, healthy cohort without hip or lumbar pathology, which may limit generalizability to older individuals with degenerative disease, deformity, prior fusion, or restricted hip motion. However, establishing normative spinopelvic relationships in an asymptomatic population is an essential first step before extrapolating to pathologic states. Additionally, motion was assessed using static radiographs obtained in standardized positions rather than real-time kinetics. Measurements were performed by a single experienced observer, and while repeated measurements were used to ensure internal consistency, formal interobserver reliability was not assessed. Finally, the reported correlations describe associations rather than causation, and the clinical implications of these quantified relationships require validation in larger and pathologic populations.

## Conclusion

In conclusion, motion between the hip, pelvis, and spine is highly correlated. When transitioning from a standing to a sitting position, the hips flex, necessitating pelvic retroversion to avoid impingement of the proximal femur on the acetabulum. The lumbar spine then flexes to maintain upright posture. For every degree increase of hip flexion, the pelvis tilts posteriorly 0.71, causing the lumbar spine to flex 1.58 degrees. For every degree increase of hip flexion, the lumbar spine decreases flexion 0.86 degrees. Given the increased interest and clinical applicability of spinopelvic biomechanics in both the arthroplasty and spine communities, these definitions are essential to moving forward with research in this topic.

## Acknowledgments

The authors received no financial support for the research, authorship, and/or publication of this article.

## Declaration of competing interest

The authors declare that they have no known competing financial interest or personal relationships that could have appeared to influence the work reported in this page.
